# In Situ Construction of a Co_2_P/CoP Heterojunction Embedded on N-Doped Carbon as an Efficient Electrocatalyst for a Hydrogen Evolution Reaction

**DOI:** 10.3390/ma17010087

**Published:** 2023-12-23

**Authors:** Ying Lei, Feng Lin, Nengyu Hong, Jian Zhang, Yulin Wang, Haijie Ben, Jianguang Li, Liyong Ding, Liang Lv

**Affiliations:** College of Chemical and Material Engineering, Quzhou University, Quzhou 324000, China; leiyingjy22@163.com (Y.L.); linfeng@qzc.edu.cn (F.L.); hny8066@163.com (N.H.); jianzhang6666@163.com (J.Z.); csu_lin@163.com (Y.W.); benhj@qzc.edu.cn (H.B.); 13867020905@163.com (J.L.)

**Keywords:** Co_2_P/CoP heterojunction, N-doped carbon, hydrogen evolution reaction, electrocatalyst

## Abstract

Noble metal-free electrocatalysts have received widespread attention in a hydrogen evolution reaction (HER) due to the importance of renewable energy development. Herein, a Co_2_P/CoP heterojunction embedded on an N-doped carbon (Co_2_P/CoP/NC) electrocatalyst was prepared via an in situ pyrolysis method. The as-prepared electrocatalyst exhibited efficient electrocatalytic activity for HER in an acidic solution. The Co_2_P/CoP/NC catalyst displayed an overpotential of 184 mV at 10 mA cm^−2^ and a low Tafel slope of 82 mV dec^−1^, which could be attributed to the tight Co_2_P/CoP heterojunction and the synergetic effect of Co_2_P/CoP and N-doped carbon. In addition, the electrochemical active surface area of Co_2_P/CoP/NC was 75.2 m^2^ g^−1^, which indicated that more active regions can be applied for the HER process. This report may pave a new way for the design of efficient and low-cost N-doped-carbon-supported 3d transition metal phosphide electrocatalysts.

## 1. Introduction

Hydrogen energy is considered as a potential alternative to fossil fuels since it has advantages of high energy density, environmental sustainability, and zero carbon emission [1,2]. Electrocatalytic splitting of water is considered as one of the viable methods for continuous large-scale hydrogen production. However, with the most active Pt-based electrocatalysts, it is difficult to achieve large-scale electrolytic water splitting application due to its high cost and low earth reserves [3,4]. Thus, the development of efficient electrocatalysts with low cost, high catalytic activity, and stability is the main challenge and key research direction in the field of an electrolytic hydrogen evolution reaction.

As an earth-abundant 3d transition metal, Fe, Co, Ni, Mn, and their compound (such as sulfides, phosphides, nitrides, carbides, and oxides) electrocatalysts have been widely studied as substitutes for noble metals [5,6,7,8]. Among them, transition metal phosphides exhibited excellent electrocatalytic hydrogen evolution activity due to the high electro-negativity of P atoms [7,9]. Particularly, cobalt phosphides, including Co_2_P and CoP, are often used as an electrocatalyst in the HER and OER process [10,11]. Previous studies have shown that the synergistic effect of Co_2_P and CoP can effectively improve the electrocatalytic activity [12,13]. Nevertheless, the synthesis methods to obtain a Co_2_P/CoP nanojunction are typically complicated as well as using NaH_2_PO_2_ as the P source [14,15,16,17], which usually require H_2_ as reduction gas or have two steps in the thermal treatment process (pyrolysis followed with the phosphating process). Therefore, one-step synthesis of a Co_2_P/CoP nanojunction using simple methods is a challenging study. In addition, transition metal phosphides tend to aggregate during the synthesis process and are easily oxidized during storage [18]. In view of this, to increase the stability of transition metal phosphides and the conductivity of the catalyst, carbon-supported nanocomposite catalysts have been developed, especially N-doped carbon materials [19]. For example, Ma et al. prepared N-doped-carbon-coated CoP nanoparticles supported on N-doped graphene through a subsequent hydrothermal treatment, pyrolysis, and a following phosphating process [18]. In another work, Chen et al. reported the preparation of CoP nanoparticles absorbed on defective reduced graphene oxide (CoP@DrGO) using NaH_2_PO_2_•H_2_O as the P source under Ar/H_2_ gas flow [11]. The synthesis process of carbon-supported cobalt phosphides typically involves multi-step reaction processes and complex control conditions. Therefore, it is imperative to find a method for synthesizing N-doped-C-supported cobalt phosphide nanomaterials. Phytic acid is an organic phosphate that can be extracted from plant seeds and has the characteristics of being green, natural, and environmentally friendly. It has been reported that it can be used as an organic phosphorus source to synthesize phosphide catalysts [7,19,20]. Polyaniline is widely used as a supporting conductive base material for catalysts due to its three-dimensional porous structure [21,22]. By adding excessive phytic acid in the synthesis process of a polyaniline precursor, the obtained polyaniline–phytic-acid can be used as a conductive carbon support material and an organic phosphorus source for the synthesis of N-atom-doped-carbon-supported metal phosphide catalysts, which can reduce catalyst aggregation and oxidation, improve conductivity, and thus improve catalytic performance. In view of these, we developed a synthesis method to prepare a N-doped-carbon-material-supported Co_2_P/CoP nanocomposite catalyst with efficient electrocatalytic performance.

Here, a Co_2_P/CoP heterojunction embedded on N-doped carbon (Co_2_P/CoP/NC) nanocomposite electrocatalysts was prepared through an in situ thermal treatment method. It should be pointed out that the synthesis process does not require additional P sources (NaH_2_PO_2_) or reducing gases (H_2_). Moreover, the obtained Co_2_P/CoP/NC catalyst exhibited an exceptional synergistic effect between Co_2_P/CoP and N-doped C, which efficiently improved its electrocatalytic hydrogen evolution performance. This research provides a method for synthesizing high-performance 3d transition metal phosphide nanocomposite electrocatalysts.

## 2. Materials and Methods

### 2.1. Materials

Aniline (C_6_H_7_N, 99+%), Ammonium persulphate ((NH_4_)_2_S_2_O_8_, 98%), and Nafion (5% *w*/*w* in water and 1-propanol) were purchased from Alfa Aesar China (Shanghai, China). The phytic acid solution (C_6_H_18_O_24_P_6_, 70% in H_2_O) was supplied by Aladdin (Shanghai, China). Cobalt (II) oxalate dihydrate (CoC_2_O_4_•2H_2_O, 99.7%) was provided from Macklin (Shanghai, China). Sulfuric acid (H_2_SO_4_, 98%) and ethanol (C_2_H_6_O, 99.7%) were supplied by Xilong Chemical Works (Shantou, China). All chemicals were used without further purification.

### 2.2. Preparation of CoC_2_O_4_/Polyaniline–Phytic-Acid (CoC_2_O_4_/PANI-PA) Precursor

The CoC_2_O_4_/PANI-PA hybrid precursor was synthesized as follows: Initially, 0.92 mL of aniline and 1.32 mL of phytic acid (70% (*w*/*w*) in H_2_O) were dissolved in 4 mL of deionized water with stirring to form a mixture. Then, 1.144 g of ammonium persulfate was dissolved in 2 mL of water to form a uniform solution. In total, 0.5 g of cobalt oxalate dihydrate and an ammonium persulfate aqueous solution were added into the mixture of aniline and phytic acid in turn, and the mixture was stirred for a certain time until the reaction was complete, forming a homogeneous mixture. We then allowed the mixture to undergo a complete polymerization reaction by standing at room temperature for 12 h. Finally, the mixture was washed and filtered with a small amount of deionized water and dried in an oven for about 12 h to obtain the CoC_2_O_4_/PANI-PA hybrid precursor material.

### 2.3. Preparation of Electrocatalysts

The one-step synthesis of the Co_2_P/CoP/NC electrocatalyst was conducted in a tube furnace. In total, 300 mg of the CoC_2_O_4_/PANI-PA hybrid precursor was pyrolyzed at 900 °C under a N_2_ atmosphere for 2 h with a ramp rate of 5 °C min^−1^. After the thermal reaction, the furnace was cooled down to room temperature and the Co_2_P/CoP embedded on N-doped carbon nanomaterial (Co_2_P/CoP/NC) was obtained. We also synthesized the samples that the CoC_2_O_4_/PANI-PA hybrid precursor pyrolyzed at different temperatures (800, 850, and 950 °C) and they were donated as Co_2_P_2_O_7_/NC, Co_2_P_2_O_7_/Co_2_P/NC, and Co_2_P/NC, respectively.

### 2.4. Structural Characterization

X-ray diffraction (XRD) patterns were examined through an X-ray diffractometer (Rigaku D/MAX-2600, Tokyo, Japan) with Cu-Kα radiation under a scanning rate of 5° min^−1^. The Raman spectrum was collected through a Raman spectrometer (HORIBA HR Evolution, Kyoto, Japan) with a 532 nm laser as the excitation light source. The fine structures and morphologies of the catalyst were characterized by a transmission electron microscope (JEOL JEM-F200, Tokyo, Japan) and scanning electron microscope (ZEISS Gemini 300, Oberkochen, Germany) equipped with energy dispersive X-ray spectra (EDS, Smartedx, Oberkochen, Germany). X-ray photoelectron spectroscopy (XPS) was examined on a Thermo Scientific K-Alpha using a monochromatic Al Kα X-ray source.

### 2.5. Electrochemical Characterization

All electrochemical measurements were examined on a three-electrode system using a Gamry Interface 1010E potentiostat (Gamry, Warminster, PA, USA). The electrochemical tests were conducted in 0.5 M H_2_SO_4_ and the catalytic electrode, Ag/AgCl electrode, and carbon rod served as a working electrode, reference electrode, and counter electrode, respectively. To prepare the catalyst ink, 4 mg of the catalysts was dispersed in the mixture of 230 μL of H_2_O, 750 μL of ethanol, and 20 μL of the Nafion solution with ultrasonic treatment for 30 min to form a uniform suspension. Then, 15 μL of the catalyst suspension was loaded onto a glassy carbon electrode (5 mm in diameter) and dried naturally in room temperature to obtain the working electrode. The polarization curves were tested using linear sweep voltammetry (LSV) with a scan rate of 5 mV s^−1^ without IR compensation in electrolytes after saturation with N_2_ for 30 min. Cyclic voltammograms (CVs) were examined in the potential range of 0.1 to 0.2 V with the scan rates from 20 to 140 mV s^−1^. All potentials in this paper were calibrated to a reversible hydrogen electrode (RHE) based on the equation E(RHE) = E(Ag/AgCl) + 0.059 pH + 0.197 V. EIS measurements were performed at −100 mV with frequency from 0.01 Hz to 100 kHz with an AC voltage of 5 mV.

## 3. Results and Discussion

**Figure 1:**
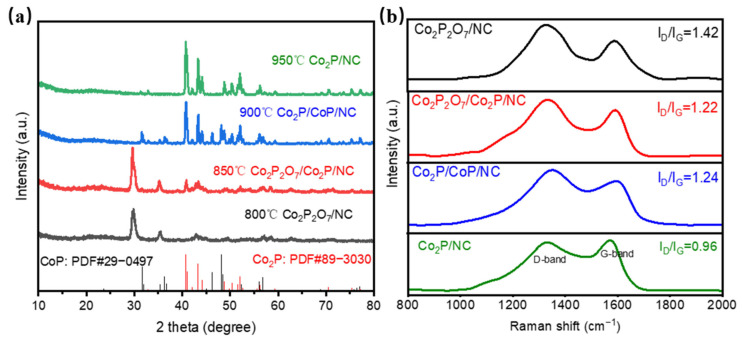
(**a**) XRD patterns of the as-prepared samples; (**b**) Raman spectra of the samples.

The Co_2_P/CoP/NC electrocatalyst was synthesized through one-step pyrolysis of CoC_2_O_4_/PANI-PA precursors. To study the growth mechanism of catalysts and synthesize high-performance catalysts, we conducted the pyrolysis process under 800, 850, 900, and 950 °C. The X-ray diffraction analysis was first performed to characterize the crystalline phase composition of the catalysts. As shown in Figure 1a, the sample obtained at 800 °C showed two obvious diffraction peaks at 29.8° and 35.3°, corresponding to the (12-2) and (130) faces of Co_2_P_2_O_7_ (JCPDS 39-0709), respectively. When the pyrolysis temperature increased to 850 °C, two additional diffraction peaks appeared at 40.8° and 43.4°, which were ascribed to the (112) and (211) diffraction peaks of Co_2_P (JCPDS 89-3030), respectively, indicating the coexistence of Co_2_P_2_O_7_ and Co_2_P. Interestingly, when the pyrolysis temperature was further increased to 900 °C, Co_2_P_2_O_7_ was completely decomposed to form a Co_2_P/CoP composite (the diffraction peaks appearing at 31.6°, 35.4°, 36.4°, 36.7°, 46.3°, 48.2°, 56.1°, and 56.8° coincide with the diffraction peaks of CoP (JCPDS 29-0497)). The XRD pattern is magnified in Figure S1 and no other diffraction peaks were found except for Co_2_P and CoP indicating there were not any byproducts in the catalyst. In addition, the diffraction peak shape of Co_2_P became sharper, indicating a higher crystallinity of the sample. It is reported that the intrinsic electrocatalytic activity of CoP is higher than that of pure Co_2_P, while Co_2_P has better conductivity during the electrocatalytic process [23]. Thus, the presence of Co_2_P can promote electron transfer performance, and the formation of a Co_2_P/CoP heterojunction can improve the electrocatalytic hydrogen evolution performance [17]. However, when the pyrolysis temperature was 950 °C (Figure S2), the diffraction peaks of CoP disappeared, indicating that 900 °C is the optimal temperature for synthesizing the Co_2_P/CoP mixed phase.

To verify the presence of carbon in the catalyst, we conducted Raman spectroscopy tests on the samples, and the result is shown in Figure 1b. Raman peaks of Co_2_P/CoP/NC located at 1349 cm^−1^ and 1593 cm^−1^ represented the disorder carbon (D-band) and graphitic carbon (G-band), respectively. The higher D band intensity indicated that more defects were generated [11,24]. Meanwhile, the intensity ratio (I_D_/I_G_) of Co_2_P_2_O_7_/NC, Co_2_P_2_O_7_/Co_2_P/NC, Co_2_P/CoP/NC, and Co_2_P/NC was 1.42, 1.22, 1.24, and 0.96, respectively. The result presented the graphitization of carbon domains formatted as the synthesis temperature increases, which can help the transfer of electrons. As a support material of transition metal phosphide, it can provide more catalytic active sites for the electrocatalytic hydrogen evolution reaction, and it is easier to adsorb hydrogen ions as well as desorb hydrogen, thereby improving the catalytic activity of the material.

**Figure 2:**
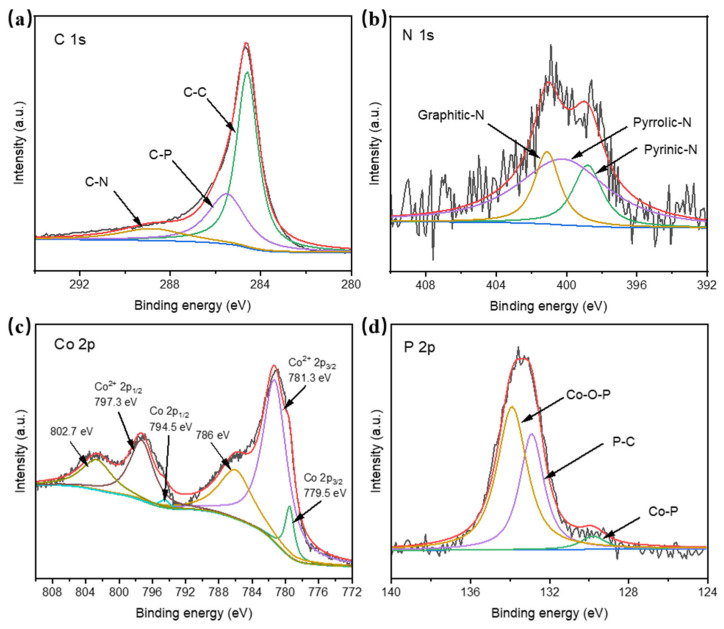
Deconvoluted XPS profiles of (**a**) C1s, (**b**) N 1s, (**c**) Co 2p, and (**d**) P 2p of Co_2_P/CoP/NC.

The surface elemental composition of Co_2_P/CoP/NC was analyzed through X-ray photoelectron spectroscopy (XPS). The full XPS survey spectrum in Figure S3 revealed the existence of C, N, O, P, and Co elements in the catalyst. The high-resolution spectrum of O 1s is shown in Figure S4 and it can be deconvoluted into two peaks at 531 and 532.4 eV, which were attributed to the zero valence state and negatively charged oxygen atoms. The presence of the oxygen element is due to the inevitable partial oxidation of the catalyst in the air. The high-resolution spectrum of C 1s resolved into three peaks located at 284.6 eV, 285.5 eV, and 288.8 eV (Figure 2a), which were attributed to C-C, C-P, and C-N, respectively [25]. The N 1s high-resolution spectrum in Figure 2b could be deconvoluted into three peaks at 398.8 eV, 400.2 eV, and 401.1 eV, which were ascribed to pyrinic-N, pyrrolic-N, and graphitic-N, respectively [26]. It has been reported that the existence of graphitic-N can greatly promote the charge transfer efficiency of a catalyst, thus improving the conductivity of the catalyst. The C 1s and N 1s spectra fitting results showed that the N element was successfully doped into the carbon matrix to form the N-doped carbon framework. The high-resolution spectrum of Co 2p for Co_2_P/CoP/NC in Figure 2c demonstrated the Co 2p_3/2_ binding energies located at 779.5 eV and 781.3 eV with one satellite peak centered at 786 eV, and the Co 2p_1/2_ binding energies located at 794.5 eV and 797.3 eV with one satellite peak at 802.7 eV [15,23,27]. The peaks located at 779.5 eV and 794.5 eV were attributed to the Co-P bonds in the Co_2_P/CoP/NC catalyst, and the peaks at 781.3 eV and 797.3 eV were distributed to the Co-O bonds, which was caused by the inevitable surface oxidation of Co_2_P/CoP. The high-resolution spectrum of P 2p in Figure 2d displayed three peaks located at 129.8 eV, 132.9 eV, and 133.9 eV that were assigned to the Co-P, P-C, and Co-O-P, respectively [28,29]. The P 2p spectrum confirmed the existence of Co-P as well as the close interaction between C and P in the Co_2_P/CoP/NC catalyst. The XPS spectra of Co_2_P_2_O_7_/NC, Co_2_P_2_O_7_/Co_2_P/NC, and Co_2_P/NC were also characterized and are illustrated in Figures S5–S9, which can further confirm the formation of Co_2_P_2_O_7_/NC, Co_2_P_2_O_7_/Co_2_P/NC, and Co_2_P/NC at different temperatures.

**Figure 3:**
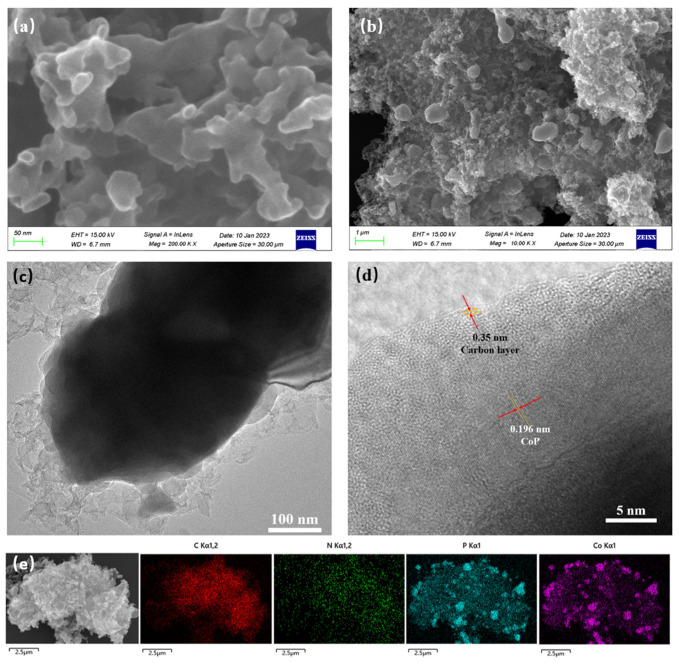
(**a**,**b**) SEM images of Co_2_P/CoP/NC; (**c**,**d**) TEM images of Co_2_P/CoP/NC at different magnifications; (**e**) EDS element mapping of Co_2_P/CoP/NC.

The morphology and nanostructure of Co_2_P/CoP/NC were studied through SEM and TEM. The SEM images in Figure 3a,b show a three-dimensional porous interconnected nanostructure with a diameter of about 100 nm. The TEM images in Figure 3c and Figure S10–S12 show the Co_2_P/CoP nanoparticles were embedded on N-doped carbon. The high-resolution TEM image in Figure 3d shows well-defined lattice fringes of 0.196 nm corresponding to the crystal plane of CoP (112). The layered structure at the edge with the lattice fringes of 0.35 nm corresponded to the (002) lattice planes of graphitic carbon [26]. The EDS spectrum of Co_2_P/CoP/NC is shown in Figure S13, and C, O, Co, and P were detected. Meanwhile, the atomic ratio of Co to P was calculated to be 1.73:1, indicating the coexistence of Co_2_P and CoP. The EDS element mapping in Figure 3e displays the C, N, Co, and P elements in the catalyst, and the element mappings of Co and P confirmed the formation of Co_2_P/CoP embedded on N-doped carbon, which is consistent with the XPS analysis.

**Figure 4:**
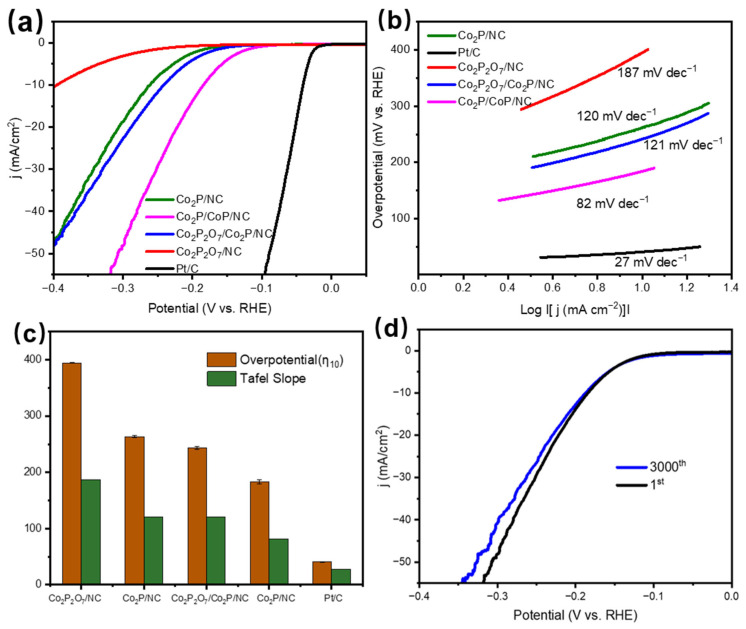
(**a**) Polarization curves and (**b**) Tafel plots of Pt/C, Co_2_P/NC, Co_2_P/CoP/NC, Co_2_P_2_O_7_/Co_2_P/NC, and Co_2_P_2_O_7_/NC, (**c**) comparison of the overpotential (η_10_) and the Tafel slope of different catalysts, (**d**) polarization curves of Co_2_P/CoP/NC before and after 3000 potential cycles.

To explore the electrocatalytic performance of the electrocatalysts, the HER was conducted in a three-electrode system, and commercial Pt/C (20 wt%) was selected as a comparison. Figure 4a shows the LSV curves of Pt/C, Co_2_P/NC, Co_2_P/CoP/NC, Co_2_P_2_O_7_/Co_2_P/NC, and Co_2_P_2_O_7_/NC at a scan rate of 5 mV/s without iR correction. Obviously, the Pt/C catalyst demonstrated the supreme HER activity with the lowest overpotential of 41 mV at a current density of 10 mA cm^−2^. Moreover, Co_2_P/CoP/NC displayed an overpotential of 184 mV at 10 mA cm^−2^, which was lower than that of Co_2_P/NC (261 mV), Co_2_P_2_O_7_/Co_2_P/NC (241 mV), and Co_2_P_2_O_7_/NC (395 mV). Through comparison, it was found that the electrocatalysts with Co_2_P and CoP coexisting had the highest HER performance, indicating the efficient catalytic activity of Co_2_P/CoP/NC. In order to further verify the optimal amount of CoC_2_O_4_•2H_2_O added for the synthesis of catalysts, we compared the HER activity of the catalysts when 0.3 g, 0.5 g, and 1 g of CoC_2_O_4_•2H_2_O were added to synthesize Co_2_P/CoP/NC at 900 °C, respectively. As shown in Figure S14, when the addition amount of CoC_2_O_4_•2H_2_O is 0.3 g, the overpotential at 10 mA cm^−2^ is 332 mV, while when the addition amount is 1 g, the overpotential is 250 mV. The lowest overpotential is found at the addition amount of 0.5 g, indicating that the optimal CoC_2_O_4_•2H_2_O addition amount is 0.5 g, which means that the optimal synthesis condition for the catalyst is at a temperature of 900 °C with a CoC_2_O_4_•2H_2_O addition of 0.5 g, under which the catalyst has the best HER performance. To better compare the HER activities, the Tafel plots fitted from the LSV curves according to the Tafel equation (η = b log (j) + a, where b is the Tafel slope) are shown in Figure 4b. Pt/C showed a Tafel slope of 27 mV dec^−1^, which is similar to the values in previous literature. The Tafel slope of Co_2_P/CoP/NC was 82 mV dec^−1^, indicating the HER behavior occurred on the Co_2_P/CoP/NC surface following a Volmer–Heyrovsky mechanism. Moreover, the Tafel slope of Co_2_P/CoP/NC was much lower than that of Co_2_P/NC (120 mV dec^−1^), Co_2_P_2_O_7_/Co_2_P/NC (121 mV dec^−1^), and Co_2_P_2_O_7_/NC (187 mV dec^−1^), suggesting faster reaction kinetics of HER on Co_2_P/CoP/NC. To estimate the stability of the Co_2_P/CoP/NC catalyst, the long-term cyclic voltammetry (CV) measurement was conducted. As shown in Figure 4d, after 3000 CV cycles, the LSV curve of Co_2_P/CoP/NC showed minimal performance degradation, indicating the stability of the catalyst. Meanwhile, we also tested the chronoamperometric response curve of Co_2_P/CoP/NC at the potential of −0.190 V (Figure S15); the result also indicated a good stability of Co_2_P/CoP/NC.

**Figure 5:**
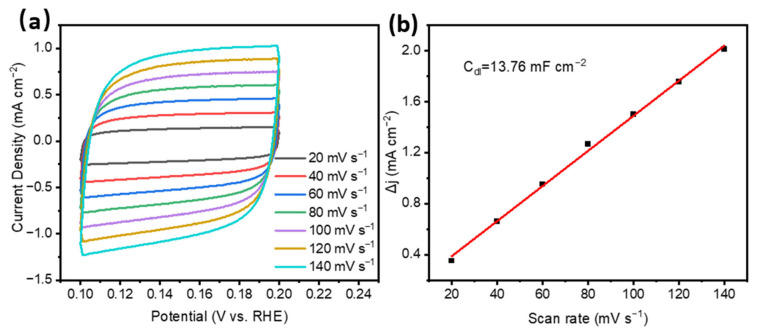
(**a**) CV curves of Co_2_P/CoP/NC under different scan rates, (**b**) extraction of electrochemical double-layer capacitance (C_dl_) through the relationship between the current density variation (Δj) and scan rate at 0.15 V versus RHE for Co_2_P/CoP/NC.

To further investigate the mechanism of enhanced electrocatalytic hydrogen evolution performance, the electrochemical double-layer capacitance (C_dl_) and electrochemical active surface area (ECSA) of Co_2_P/CoP/NC were derived by testing the CV curves at different scan rates (Figure 5a). The C_dl_ was obtained by linearly fitting Δj (Δj = j_a_ − j_c_) at 0.15 V with the scanning rate in Figure 5b, and the C_dl_ of Co_2_P/CoP/NC was 13.76 mF cm^−2^, which is comparable compared to other cobalt phosphorus compound catalysts, such as Co_2_P/CoP (0.434 mF cm^−2^) [15], Co_2_P/CoMoP_x_/NF (11.1 mF·cm^−2^) [27], and CoP/NiCoP/NC (19.3 mF cm^−2^) [6]. The ECSA of Co_2_P/CoP/NC was calculated in Figure S16 according to the C_dl_, and the ECSA was 75.2 m^2^/g by assuming a standard value of 60 μF/cm^2^. The large ECSA of Co_2_P/CoP/NC indicates more active regions for the electrocatalytic hydrogen evolution process.

**Figure 6:**
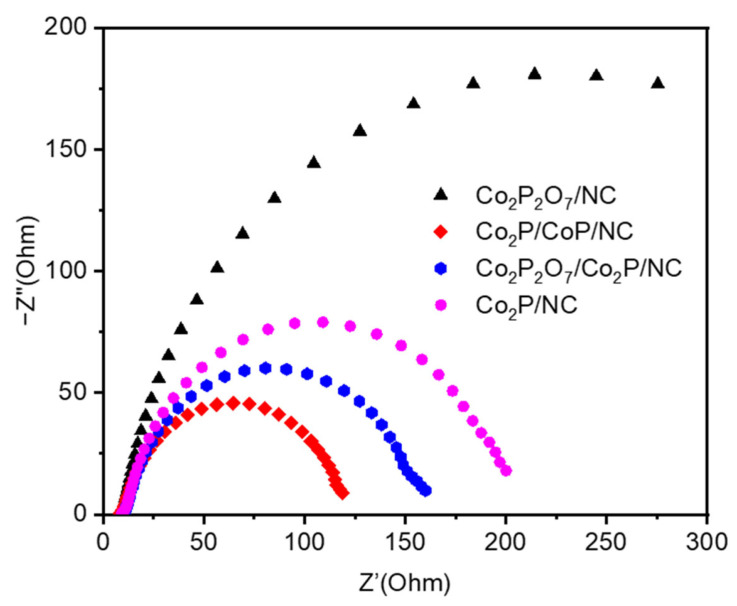
Nyquist plots of Co_2_P_2_O_7_/NC, Co_2_P_2_O_7_/Co_2_P/NC, Co_2_P/CoP/NC, and Co_2_P/NC at the potential of −100 mV (vs. RHE).

The EIS was performed to investigate the catalytic kinetics for HER, and Nyquist plots are shown in Figure 6. Co_2_P/CoP/NC presents a smaller semicircle of the Nyquist plot, which means a smaller R_ct_ and a faster electron transfer ability of the catalyst. According to the characterization results and the electrochemical studies, the electrocatalytic performance of the Co_2_P/CoP/NC catalyst mainly originated from the following aspects: (1) The Co_2_P/CoP heterojunction formed through the combination of CoP with high intrinsic electrocatalytic activity and Co_2_P with good conductivity, endowing the catalyst with high electrocatalytic activity. (2) The presence of defective carbon supporting materials was beneficial to the adsorption and desorption of H^+^ and H_2_, thereby improving the electrocatalytic performance. Meanwhile, the formation of N-doped C could provide a better charge transfer efficiency, resulting in high conductivity for the catalyst. (3) The high ECSA indicates that the catalyst can provide more reactive active regions and synergistic effect between Co_2_P, CoP, and NC, significantly promoting the electrocatalytic hydrogen evolution performance of the catalyst.

## 4. Conclusions

In this study, we successfully synthesized a Co_2_P/CoP heterojunction embedded on N-doped carbon nanocomposites through a pyrolysis method. The formation of Co_2_P/CoP heterojunctions contributes to the improvement in electrocatalytic performance, and the presence of N-doped-defective-C-supported materials helps to boost the conductivity of the catalyst and the adsorption/desorption of H^+^ and H_2_, while the close contact between Co_2_P/CoP and N-doped C enables Co_2_P/CoP/NC to possess efficient electrocatalytic performance. Consequently, the Co_2_P/CoP/NC electrocatalyst exhibits efficient electrocatalytic hydrogen evolution ability, with an overpotential of 184 mV at 10 mA cm^−2^, a Tafel slope of 82 mV dec^−1^, and remarkable long-term stability. This work provides a method for synthesizing N-doped-carbon-supported iron, cobalt, and nickel phosphide nanocomposite electrocatalysts.

## Data Availability

Data are contained within the article and Supplementary Materials.

## Supplementary Materials

The following supporting information can be downloaded at: https://www.mdpi.com/article/10.3390/ma17010087/s1, Figure S1. XRD pattern of Co_2_P/CoP/NC; Figure S2. XRD pattern of Co_2_P/NC; Figure S3. XPS survey spectrum of Co_2_P/CoP/NC; Figure S4. Deconvoluted XPS profiles of O 1s of Co_2_P/CoP/NC; Figure S5. C 1s XPS spectra of Co_2_P_2_O_7_/NC, Co_2_P_2_O_7_/Co_2_P/NC, and Co_2_P/NC; Figure S6. N 1s XPS spectra of Co_2_P_2_O_7_/NC, Co_2_P_2_O_7_/Co_2_P/NC, and Co_2_P/NC; Figure S7. Co 2p XPS spectra of Co_2_P_2_O_7_/NC, Co_2_P_2_O_7_/Co_2_P/NC, and Co_2_P/NC; Figure S8. P 2p XPS spectra of Co_2_P_2_O_7_/NC, Co_2_P_2_O_7_/Co_2_P/NC, and Co_2_P/NC; Figure S9. O 1s XPS spectra of Co_2_P_2_O_7_/NC, Co_2_P_2_O_7_/Co_2_P/NC, and Co_2_P/NC; Figure S10. TEM image of Co_2_P/CoP/NC; Figure S11. TEM image of Co_2_P/CoP/NC; Figure S12. TEM image of Co_2_P/CoP/NC; Figure S13. EDS spectrum of Co_2_P/CoP/NC; Figure S14. Polarization curves of the electrocatalysts synthesized at 900 °C with different addition of CoC_2_O_4_•2H_2_O (0.3g, 0.5g and 1g); Figure S15. Chronoamperometric response at the potential of -0.190 V vs. the RHE; Figure S16. The calculation of ECSA for Co_2_P/CoP/NC.
