# Supplementary material for: In Situ Construction of a Co2P/CoP Heterojunction Embedded on N-Doped Carbon as an Efficient Electrocatalyst for a Hydrogen Evolution Reaction
Source: Materials (Basel). 2023 Dec 23;17(1):87. doi: 10.3390/ma17010087 (PMC10780241; doi:10.3390/ma17010087)
Supplement: Supplementary file 1 [file materials-17-00087-s001.zip › materials-2744391-supplementary.pdf]

Supporting Information

In Situ Construction of a Co_2P/CoP Heterojunction Embedded on N-Doped Carbon as an Efficient Electrocatalyst for a Hydrogen Evolution Reaction

Ying Lei, Feng Lin, Nengyu Hong, Jian Zhang, Yulin Wang, Haijie Ben, Jianguang Li, Liyong Ding *
and Liang Lv *

College of Chemical and Material Engineering, Quzhou University, Quzhou 324000, China; leiyingjy22@163.com (Y.L.); linfeng@qzc.edu.cn (F.L.); hny8066@163.com (N.H.); jianzhang6666@163.com (J.Z.); csu_lin@163.com (Y.W.); benhj@qzc.edu.cn (H.B.); 13867020905@163.com (J.L.)

* Correspondence: liyongding1988@163.com (L.D.); lianglv_buct@126.com (L.L.)

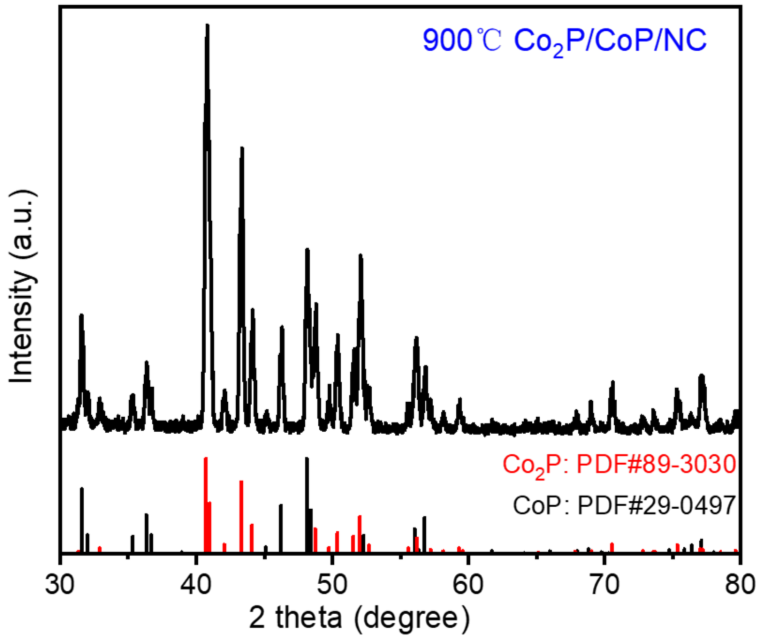

Figure S1. XRD pattern of Co_2P/CoP/NC.

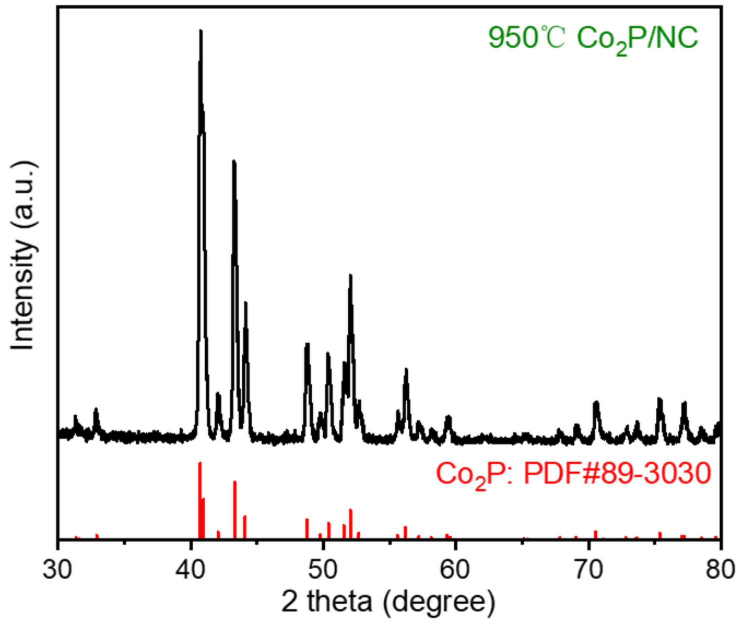

Figure S2. XRD pattern of Co_2P/NC.

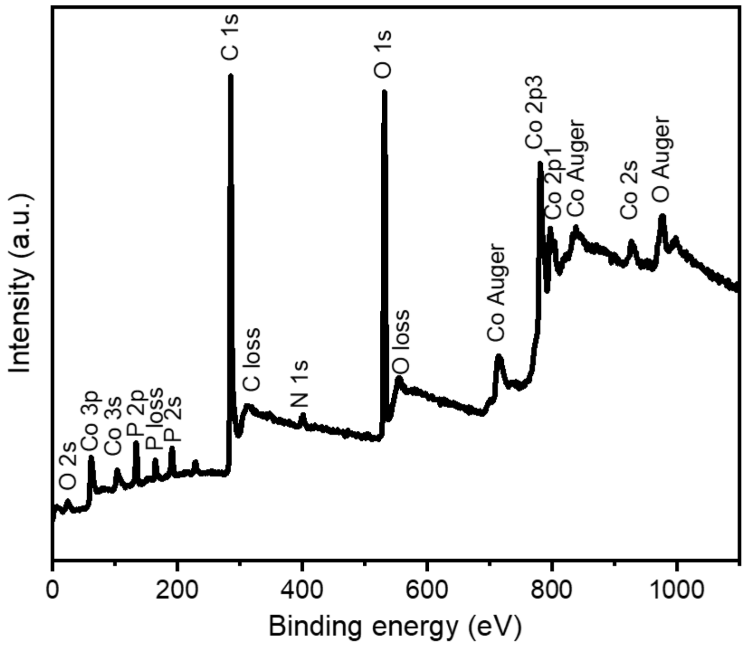

Figure S3. XPS survey spectrum of Co_2P/CoP/NC.

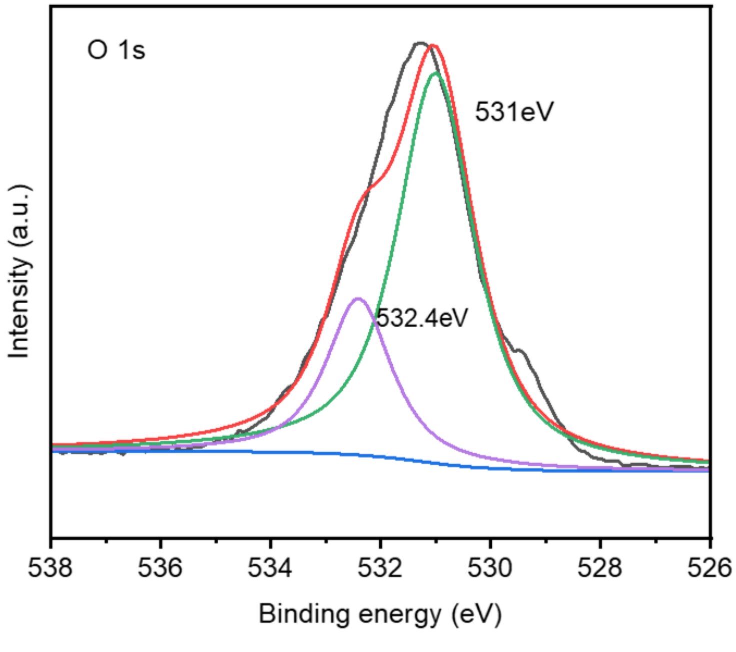

Figure S4. Deconvoluted XPS profiles of O 1s of Co_2P/CoP/NC.

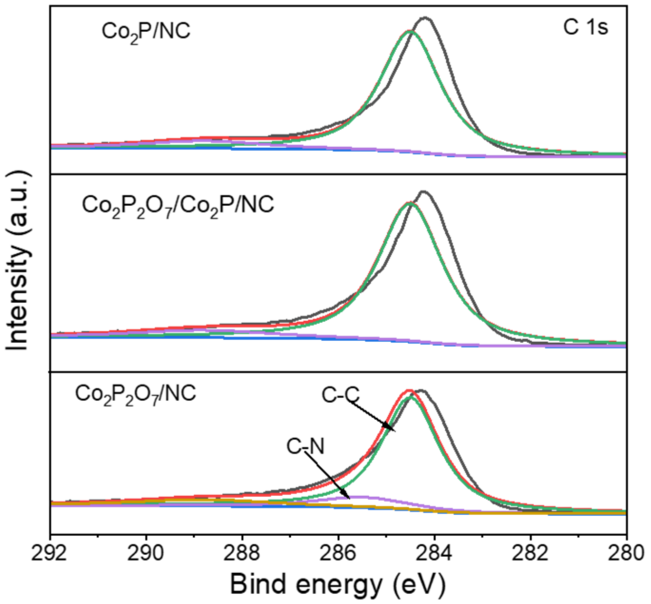

Figure S5. C 1s XPS spectra of $Co_2P_2O_7$/NC, $Co_2P_2O_7$/Co_2P/NC, and Co_2P/NC.

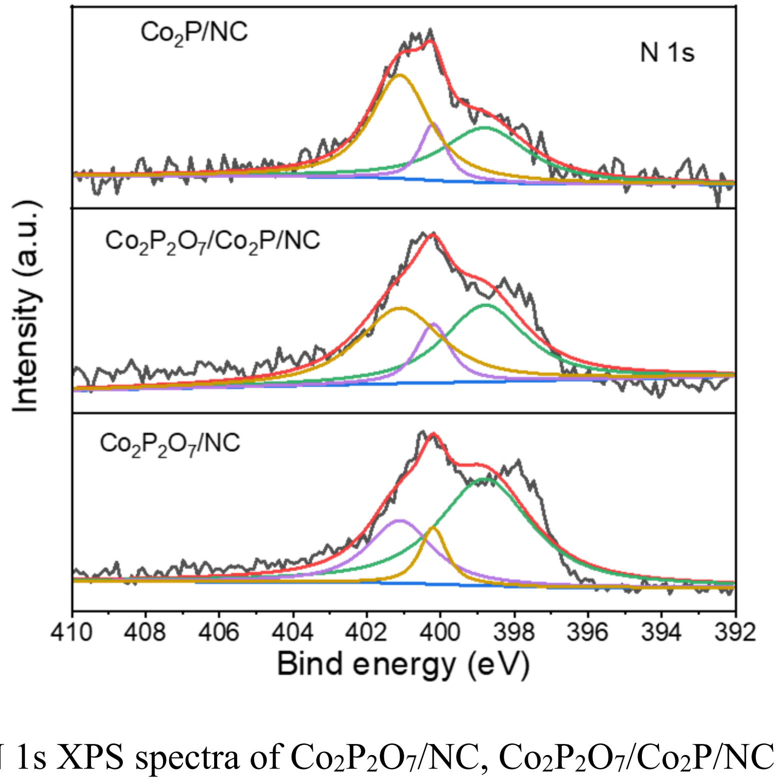

Figure S6. N 1s XPS spectra of $Co_2P_2O_7$/NC, $Co_2P_2O_7$/Co_2P/NC, and Co_2P/NC.

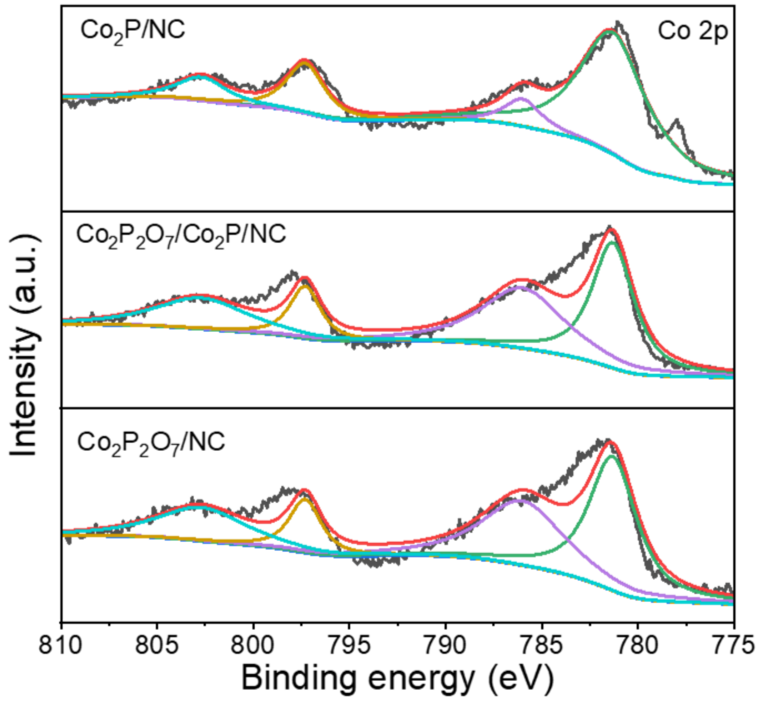

Figure S7. Co 2p XPS spectra of $Co_2P_2O_7$/NC, $Co_2P_2O_7$/Co_2P/NC, and Co_2P/NC.

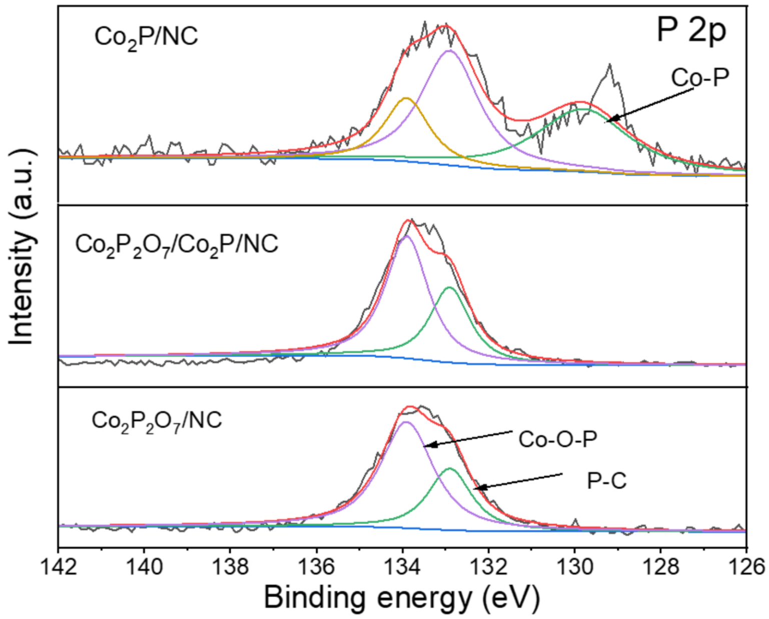

Figure S8. P 2p XPS spectra of $Co_2P_2O_7$/NC, $Co_2P_2O_7$/Co_2P/NC, and Co_2P/NC.

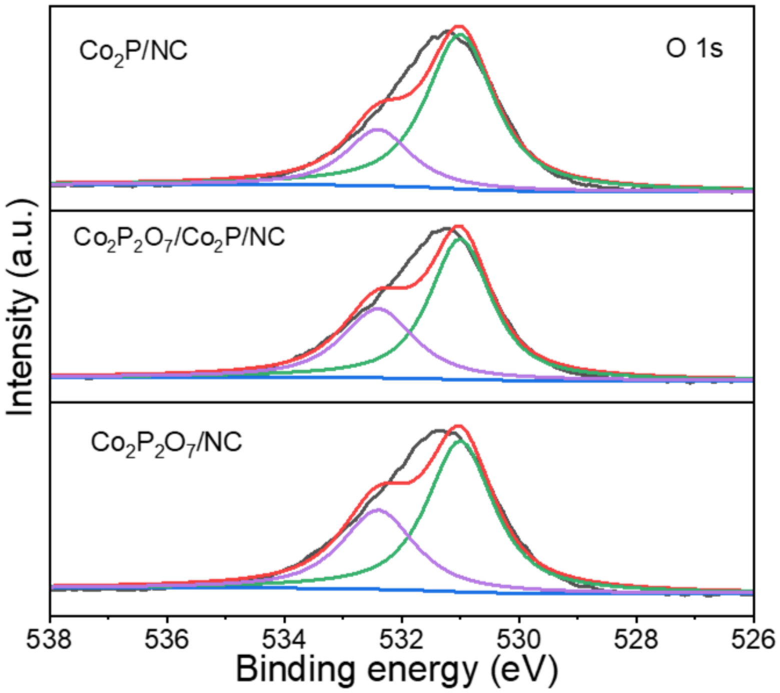

Figure S9. O 1s XPS spectra of $Co_2P_2O_7$/NC, $Co_2P_2O_7$/Co_2P/NC, and Co_2P/NC.

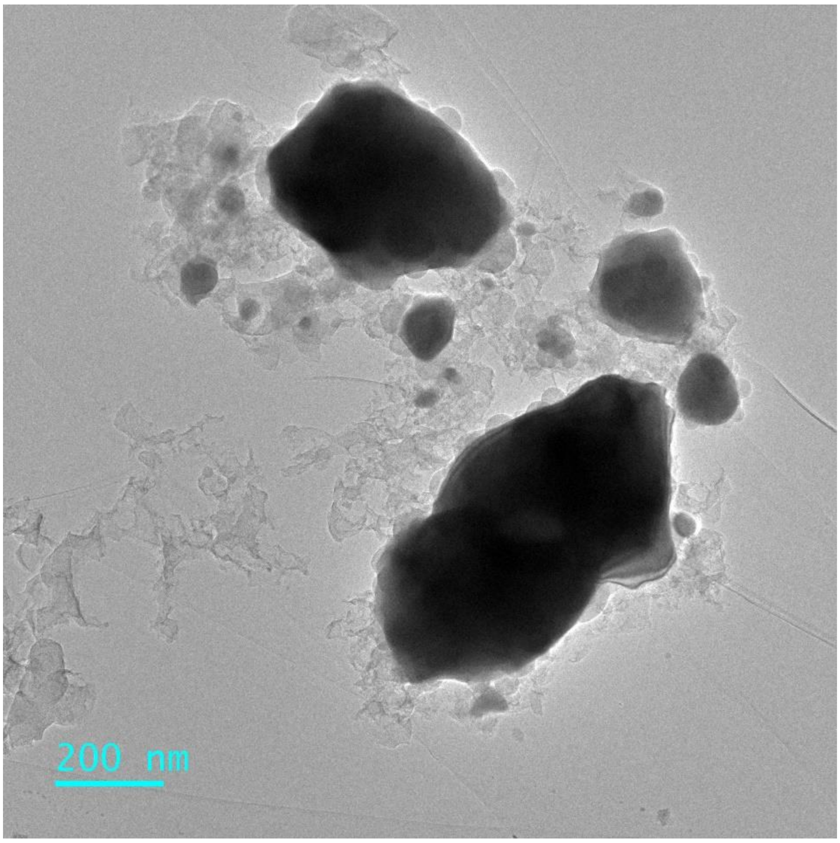

Figure S10. TEM image of Co_2P/CoP/NC.

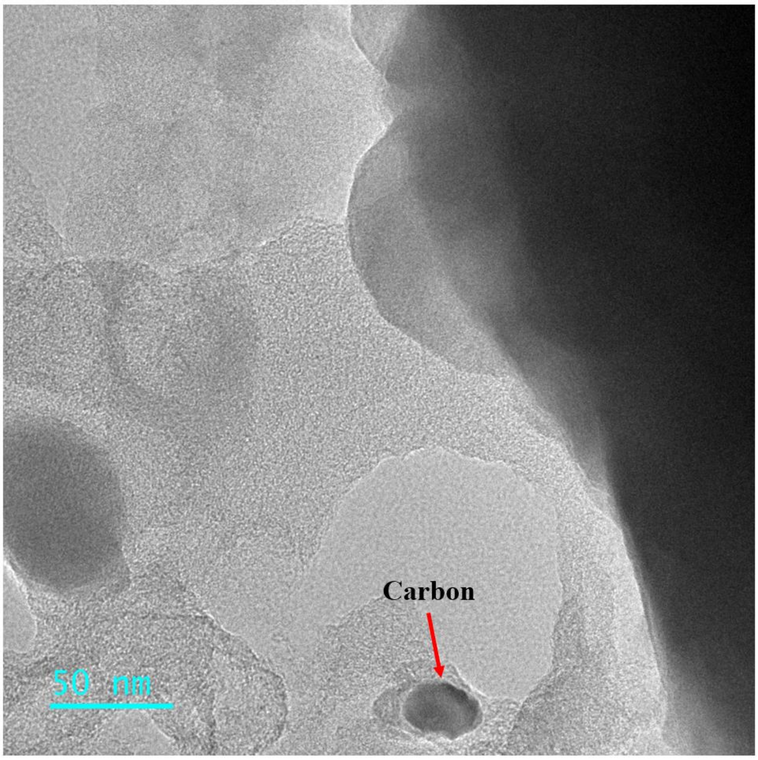

Figure S11. TEM image of Co_2P/CoP/NC.

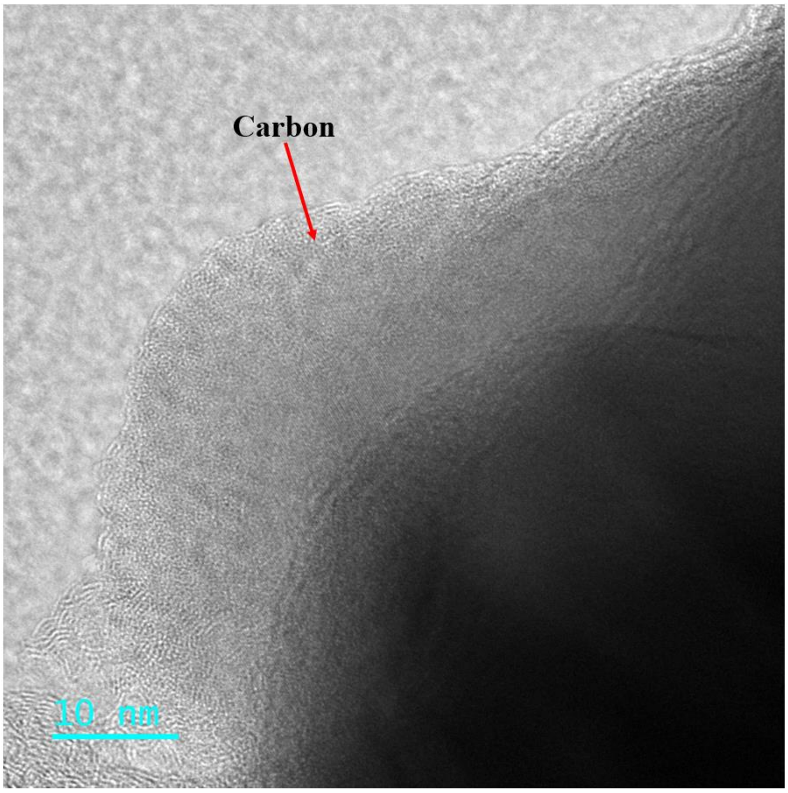

Figure S12. TEM image of Co_2P/CoP/NC.

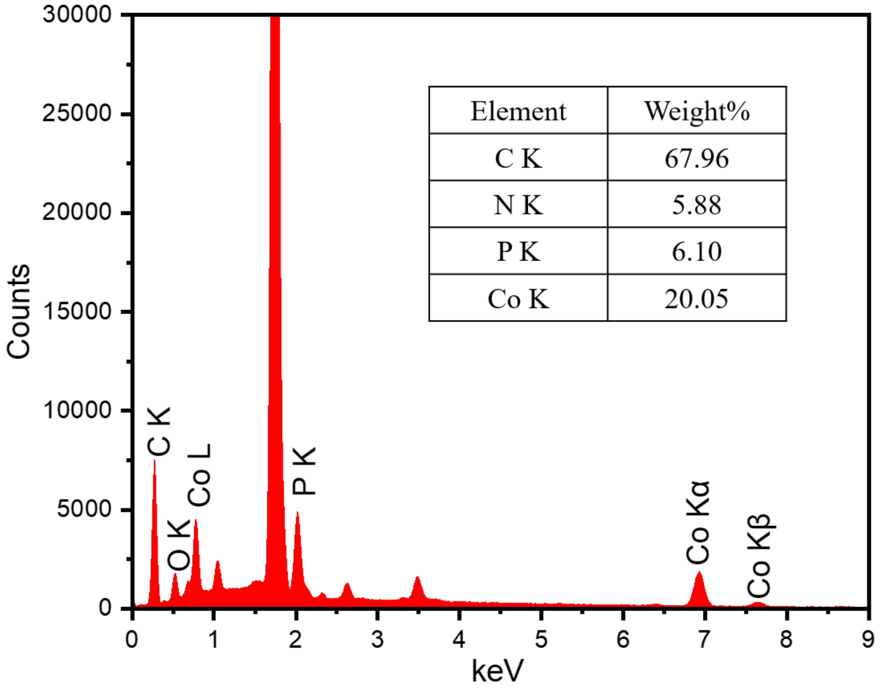

Figure S13. EDS spectrum of $Co_2P/CoP/NC$.

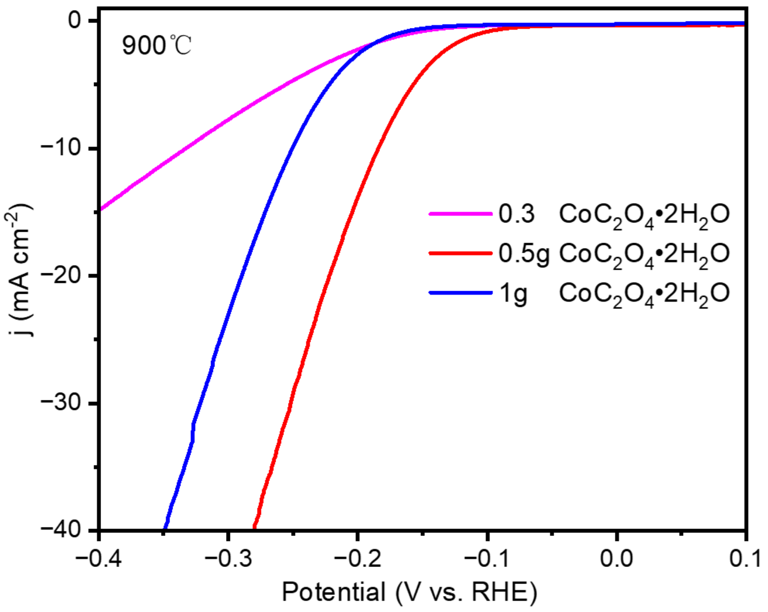

Figure S14. Polarization curves of the electrocatalysts synthesized at 900℃ with different additions of $CoC_2O_4 \cdot 2H_2O$ (0.3g, 0.5g, and 1g).

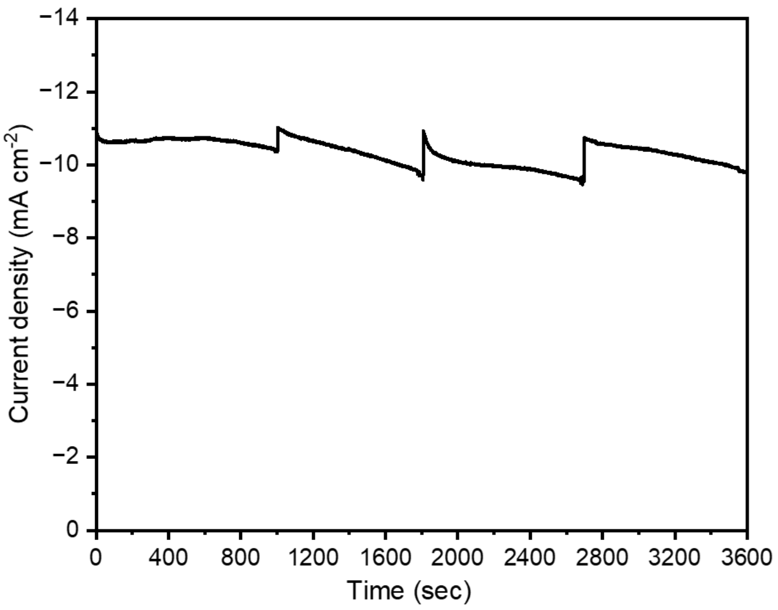

Figure S15. Chronoamperometric response at the potential of -0.190 V vs. the RHE.

Loading amount of the catalyst is 0.305 mg cm^{-2}, we assume a standard value of 60 μF/cm^2, and C_{dl} of the catalyst is 13.76 mF cm^{-2}.

C = C_{dl}/m = 45.11 F/g

ECSA= C/(60 μF cm^{-2}) = 75.2 m^2/g

Figure S16. The calculation of ECSA for Co_2P/CoP/NC.
